# Axial Impact Response of Carbon Fiber-Reinforced Polymer Structures in High-Speed Trains Based on Filament Winding Process

**DOI:** 10.3390/ma17204970

**Published:** 2024-10-11

**Authors:** Aiqin Tian, Kang Sun, Quanwei Che, Beichen Jiang, Xiangang Song, Lirong Guo, Dongdong Chen, Shoune Xiao

**Affiliations:** 1 School of Materials Science and Engineering, Shanghai Jiao Tong University, Shanghai 200240, China; tianaiq@163.com (A.T.); ksun@sjtu.edu.cn (K.S.); 2 CRRC Qingdao Sifang Co., Ltd., Qingdao 266111, China; chequanwei@cqsf.com (Q.C.); jiangbeichen.1215@163.com (B.J.); songxiangang@cqsf.com (X.S.); 3 Locomotive & Car Research Institute, China Academy of Railway Sciences Co., Ltd., Beijing 100081, China; jd_glr@163.com; 4 State Key Laboratory of Rail Transit Vehicle System, Southwest Jiaotong University, Chengdu 610031, China; cdd-2021@swjtu.edu.cn

**Keywords:** high-speed train, energy-absorbing tube, crashworthiness, CFRP, energy absorption

## Abstract

The continuous increase in the operating speed of rail vehicles demands higher requirements for passive safety protection and lightweight design. This paper focuses on an energy-absorbing component (circular tubes) at the end of a train. Thin-walled carbon fiber-reinforced polymer (CFRP) tubes were prepared using the filament winding process. Through a combination of sled impact tests and finite element simulations, the effects of a chamfered trigger (Tube I) and embedded trigger (Tube II) on the impact response and crashworthiness of the structure were investigated. The results showed that both triggering methods led to the progressive end failure of the tubes. Tube I exhibited a mean crush force (MCF) of 891.89 kN and specific energy absorption (SEA) of 38.69 kJ/kg. In comparison, the MCF and SEA of Tube II decreased by 21.2% and 21.9%, respectively. The reason for this reduction is that the presence of the embedded trigger in Tube II restricts the expansion of the inner plies (plies 4 to 6), thereby affecting the overall energy absorption mechanism. Based on the validated finite element model, a modeling strategy study was conducted, including the failure parameters (DFAILT/DFAILC), the friction coefficient, and the interfacial strength. It was found that the prediction results are significantly influenced by modeling methods. Specifically, as the interfacial strength decreases, the tube wall is more prone to circumferential cracking or overall buckling under axial impact.

## 1. Introduction

Thin-walled tube structures offer advantages such as stable energy absorption and ease of manufacturing and assembly [1,2,3], resulting in their wide use in the passive safety protection systems of transportation equipment such as rail vehicles [4,5,6], automobiles [7,8,9], and helicopters [10]. Taking rail vehicles as an example, these structures can absorb impact kinetic energy through stable deformation and failure, thus ensuring the safety of passengers during impact accidents. Extensive research has been conducted on energy-absorbing structures based on metal materials [11]. In recent years, fiber-reinforced composites, especially carbon fiber-reinforced polymer (CFRP), have gained widespread attention in energy-absorbing structure design due to their excellent mechanical properties and lightweight potential [12,13,14]. Numerous studies have shown that, compared to traditional metal energy-absorbing structures, CFRP structures can enhance lightweight performance while maintaining protection capabilities [15,16,17].

In recent years, researchers have conducted experimental and numerical studies on the crashworthiness of CFRP structures with different design parameters, such as cross-sectional shapes [18], fiber types [19,20], triggering methods [21,22], and filling materials [23,24]. Mamalis et al. [25] identified three failure modes of CFRP circular tubes under axial loading—progressive end failure, mid-span fracture, and mixed failure—with the progressive end failure mode offering the best crashworthiness. Strohrmann et al. [26] prepared carbon fiber, flax, and hybrid laminate composite tubes, and conducted quasi-static and dynamic impact tests (8 m/s). They found that the energy absorption of hybrid tubes was only 15% less than that of CFRP tubes, and the impact response of carbon/flax hybrid and flax tubes was insensitive to impact velocity. Xiao et al. [27] found that increasing the number of plies in hat-shaped CFRP structures led to greater fluctuations in the quasi-static crushing force curve.

Compared to the prepreg winding or molding process, the preparation of CFRP tubes based on the filament winding process is more efficient. However, there is limited research on its crashworthiness. Ryzińska et al. [28] studied the axial crushing behavior of unidirectional and woven fabric hybrid structures, and found that increasing the proportion of unidirectional fibers from 0.5 to 1 led to a specific energy absorption (SEA) increase of more than 35%, and that under dynamic impact conditions, the SEA of the structure decreased by more than 10% compared to that under quasi-static conditions. Özbek et al. [29] investigated the axial crushing performance of composite tubes with fiber orientation angles [±40°/±55°/±70°], and reported that when the winding angle was small, inter-ply cracks propagated longitudinally to form longer cracks, while larger winding angles reduced the length of inter-ply cracks. Wang et al. [30] found that as the winding angle increased from ±15° to ±70°, the average force of the composite tube under quasi-static and dynamic conditions decreased by 40.0% and 23.6%, respectively.

Finite element simulations can reduce testing time and costs, and finite element software packages such as ABAQUS [31,32,33], LS-DYNA [34,35], and PAM-CRASH [36,37] have been widely used in the study of the impact response of CFRP structures. However, due to the complex nonlinear deformation and failure characteristics of materials and structures during impact, further research is needed in this area. Reiner et al. [38] established crushing models of composite laminates and circular tubes based on the ABQ_DLR_UD material model in ABAQUS/Explicit, and the MAT89 and MAT219 material models in LS-DYNA. The results showed that (1) the commonly used crack band scaling in these continuum damage mechanics-based material models is not suitable for crush simulations where material fragmentation is the energy dissipation mechanism; (2) increasing mesh density reduces the stability of impact force prediction; and (3) structured meshes may cause excessive peak forces. For composite structure simulations, a multi-layer shell modeling strategy has been proposed and widely validated. This strategy achieves interlayer and intralayer deformation behavior by constructing multi-layer shell elements and introducing interlayer cohesion/contacts. Chen et al. [39] established a finite element model using ABAQUS/Explicit and validated it through axial crushing tests. A comparison of simulation and experimental results revealed that parameters such as the number of shell element layers, the friction coefficient, and the interface performance significantly affect the prediction results. Under quasi-static loading, the energy absorption mechanism of CFRP tubes includes the damage of the tube wall materials and friction with the rigid wall, accounting for 69.1% and 24.1%, respectively, of the total energy.

Although there have been numerous experimental and numerical simulation studies on CFRP energy-absorbing structures, most have focused on the automotive and aerospace fields, where the structure thickness usually does not exceed 6 mm, and CFRP structures in these studies have rarely been prepared using the winding process. In contrast, due to the characteristics of large mass and high impact energy, the energy-absorbing structures in railway vehicles generally have a wall thickness exceeding 10 mm. The winding process has garnered widespread attention due to its advantages of high automation and high raw material utilization (low process costs). However, existing studies [40,41] have shown that the mechanical properties of composite structures with wall thicknesses greater than 6 mm differ significantly from those of thin-walled plates. As the wall thickness increases, the research methods applicable to small-sized energy-absorbing structures are no longer suitable.

The rest of this paper is organized as follows. Section 2 describes the preparation of two types of CFRP specimens, based on a chamfered trigger and an embedded trigger, and introduces the dynamic impact test methods and performance evaluation indicators. Section 3 describes the numerical modeling methods based on LS-DYNA software. In Section 4, the dynamic impact test results of CFRP tubes, including the load–displacement curves, failure processes, and crashworthiness, are analyzed, and we describe the mechanism by which the trigger devices affect the crashworthiness of the structures, based on the validation of the accuracy of the numerical models. Section 5 discusses the sensitivity analysis of key modeling parameters, including failure parameters, friction coefficients, and interfacial strength.

## 2. Experimental Testing

### 2.1. Specimen Preparation

**Figure 1:**
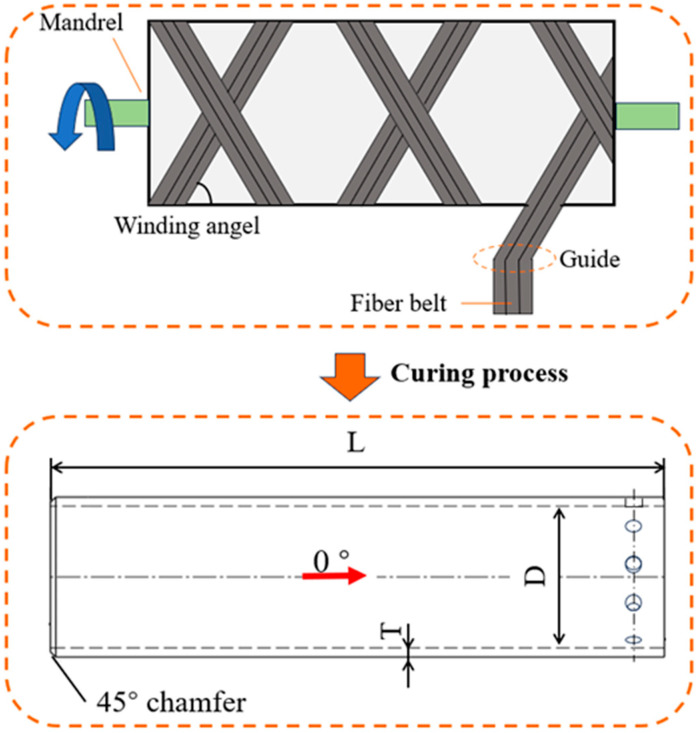
Preparation of composite material tubes.

The composite specimens were prepared at CRRC Qingdao Sifang Co., Ltd., Qingdao, China. Using T700 carbon fibers as raw materials, CFRP circular tube specimens were prepared using the winding process (Figure 1). After heating, pressurizing, and curing, the composite material tubes were cooled and demolded. The fiber winding angle was [(±5°/±5°/±88°)_15_]. Each tube had a length (*L*) of 914 mm, an inner diameter (*D*) of 220 mm, and a thickness (*T*) of 18 mm (Table 1).

### 2.2. Experimental Method

**Figure 2:**
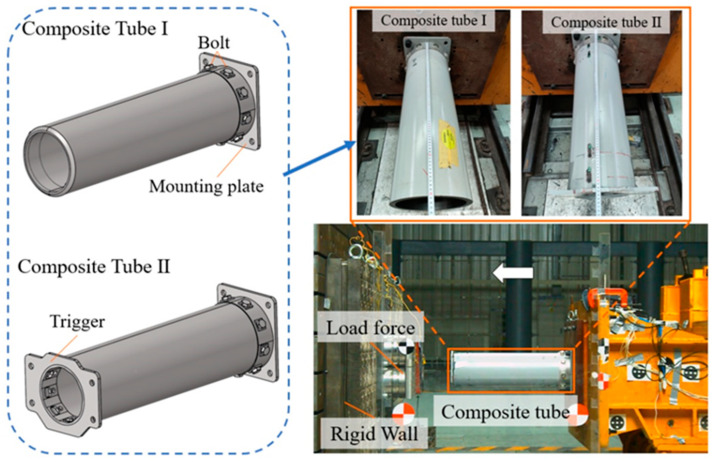
Dynamic impact test.

Dynamic impact tests were performed at CRRC Qingdao Sifang Co., Ltd., Qingdao, China. Figure 2 shows the setup, which included a sled, rigid wall, rails, force sensor, and high-speed camera. The sled had a counterweight of 12.64 t, and the mounting plate of the CFRP tube was connected to the sled using four M20 bolts. After the sled accelerated to the impact speed, it impacted the rigid wall. The load data were obtained by force sensors fixed to the rigid wall, and the high-speed camera captured the structural deformation of the specimen. Displacement data were also obtained by calibrating the captured video. Two triggering methods were considered in this test: Tube I had only a 45° chamfer on the impact end, while Tube II had a trigger device in addition to this. The trigger device was designed as an embedded cover-pressed structure and was connected to the end of the tube using bolts.

Since no external protective devices were adopted, the kinematic energy of the sled had to be absorbed through the structural deformation of the tested tubes. The impact velocity for Tube I was trial set as 24.83 km/h to avoid direct contact between the sled and the rigid wall. In the following test, the impact velocity was increased to 31.8 km/h. This velocity was close to 36 km/h, which was required in the crashworthiness evaluation of railway vehicle structures according to the EN 15227 standard [42]. The differences in impact velocity showed negligible influences on the crashworthiness evaluation of CFRP energy-absorbing structures according to conclusions from previous studies [20,25].

### 2.3. Energy Absorption Performance Indicators

The crashworthiness of CFRP energy-absorbing tubes was evaluated using the mean crushing force (MCF), energy absorption (EA), and SEA as indicators, which can all be calculated from the load–displacement curve obtained from experimental testing.

EA refers to the impact energy absorbed by the CFRP tube throughout the crushing process:

(1) $$\mathrm{EA}=\int_{0}^{d}F(x)dx$$

where *F*(*x*) is the crushing force at displacement *x* and *d* is the maximum crushing distance of the energy-absorbing tube at the end of the impact. MCF is the average crushing force,

(2) $$\mathrm{MCF}=\frac{EA}{d}$$

and SEA is the energy absorption capacity of the material per unit mass,

(3) $$\mathrm{SEA}=\frac{\mathrm{EA}\times L}{m\times d}$$

where *m* is the total mass of the specimen.

## 3. Numerical Simulation

### 3.1. FE Model

**Figure 3:**
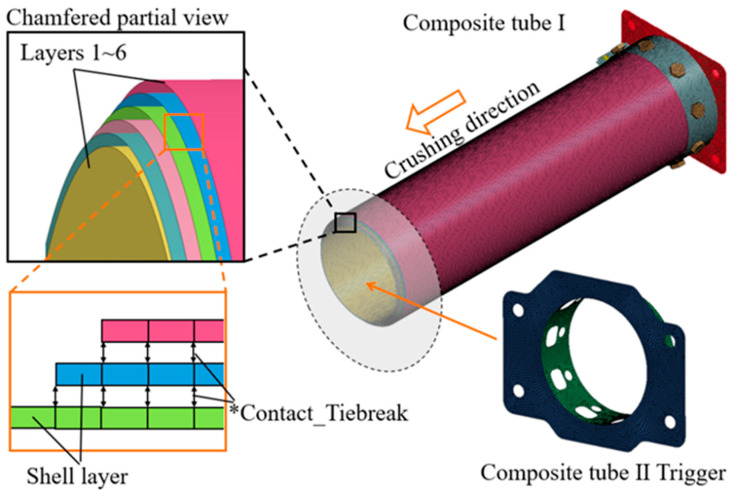
Numerical model for axial crushing of CFRP tubes.

LS-DYNA finite element (version 7.1) software was used to establish a finite element model for the axial impact response of composite tubes, as shown in Figure 3. The model consists of the specimen, the rigid wall, the bottom mounting structure, and the trigger device. Using the classical multi-layer shell strategy, the 90 winding layers of the composite tube were simulated with 6 shell elements with a cell size of 4 × 4 mm. Tiebreak contact was introduced between adjacent layers to simulate the inter-ply behavior of the composite material. A 45° chamfer on the structure was simulated by setting various layer length differences. The trigger device and fixed pressure plate were modeled using the MAT24 bilinear material model, and the rigid wall was modeled using the MAT20 rigid material model.

The bottom mounting plate retained only the degree of freedom in the impact direction, and the sled was simplified to a mass point, with a mass of 12.64 tons. Consistent with the experimental boundary conditions, the corresponding impact speed of the sled was applied to both the mounting plate and the specimen. The keyword *CONTACT_AUTOMATIC was introduced to simulate the contact between the tube wall and the rigid wall during the impact process, and *CONTACT_SELF represented potential self-contact. The dynamic and static friction coefficients were set to 0.2 and 0.15, respectively.

### 3.2. In-Plane Constitutive Model

The composite material was simulated using the MAT54 material model, which can simulate the orthotropic behavior of shell elements. The Chang–Chang failure criterion is applied as follows.

Fiber in tension:

(4) $$\sigma_{aa}>0,e_{f}^{2}=(\frac{\sigma_{aa}}{X_{t}}{)}^{2}+\beta (\frac{\sigma_{ab}}{S_{c}}{)}^{2}-1\left\{\begin{array}{c} \geq 0\;\mathrm{failed}\\ <0\;\mathrm{elastic} \end{array}\right.$$

(5) $$E_{a}=E_{b}=G_{ab}=\nu_{ab}=\nu_{ba}=0$$

where *a* and *b* represent the fiber direction and the direction perpendicular to the fiber in the plane, respectively, i.e., the longitudinal and transverse directions; $\nu_{ab}$ is Poisson’s ratio; and $E_{a}$, $E_{b}$, and $G_{ab}$, respectively, are the elastic moduli in the *a* and *b* directions and the shear modulus of the material.

Fiber in compression:

(6) $$\sigma_{aa}<0,e_{c}^{2}=(\frac{\sigma_{aa}}{X_{c}}{)}^{2}-1\left\{\begin{array}{c} \geq 0\;\mathrm{failed}\\ <0\;\mathrm{elastic} \end{array}\right.$$

(7) $$E_{a}=\nu_{ab}=\nu_{ba}=0$$

Matrix in tension:

(8) $$\sigma_{bb}>0,e_{m}^{2}=(\frac{\sigma_{bb}}{Y_{t}}{)}^{2}+(\frac{\sigma_{ab}}{S_{c}}{)}^{2}-1\left\{\begin{array}{c} \geq 0\;\mathrm{failed}\\ <0\;\mathrm{elastic} \end{array}\right.$$

(9) $$E_{b}=\nu_{ba}=0\to G_{ab}=0$$

Matrix in compression:

(10) $$\sigma_{bb}<0,e_{d}^{2}=(\frac{\sigma_{bb}}{2S_{c}}{)}^{2}+\left[(\frac{Y_{c}}{2S_{c}}{)}^{2}-1\right](\frac{\sigma_{bb}}{Y_{c}})+(\frac{\sigma_{ab}}{S_{c}}{)}^{2}-1\left\{\begin{array}{c} \geq 0\;\mathrm{failed}\\ <0\;\mathrm{elastic} \end{array}\right.$$

(11) $$E_{b}=\nu_{ab}=\nu_{ba}=0\to G_{ab}=0.$$

The mechanical properties and failure parameters of the CFRP material model are shown in Table 2 and Table 3, respectively, with the parameters in Table 2 provided by the manufacturer. The failure parameters in Table 3 generally have a significant effect on the prediction results, but these parameters do not have a clear physical meaning and are difficult to obtain through specific material tests. Therefore, those failure parameters refer to Refs. [43,44], where the similar materials were used. The mechanical parameters of the Q355 material are shown in Table 4, as provided by the manufacturer.

### 3.3. Interlayer Model

**Figure 4:**
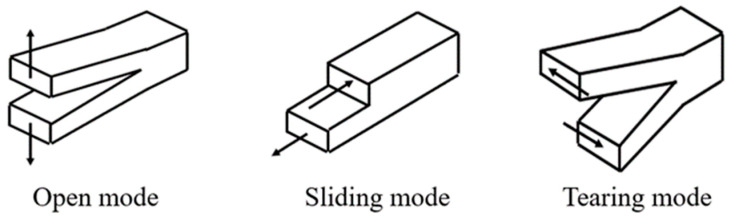
Schematic of delamination failure mode.

Delamination is a failure mode that is widespread in the crushing process of composite structures. Depending on the loading conditions, delamination can be categorized into opening mode (Mode I, subjected to tensile loading perpendicular to the crack surface), sliding mode (Mode II, subjected to shear loading perpendicular to the crack front), and tearing mode (Mode III, subjected to shear loading parallel to the crack front), as shown in Figure 4. To simulate delamination between different plies of circular tubes during impact, the contact between adjacent shell element layers is defined using *CONTACT_TIEBREAK, and various failure criteria are selected through the Option parameter to meet the requirements of different element types. The stress failure criteria are as follows:

(12) $$(\frac{\left|\sigma_{n}\right|}{NFLS}{)}^{2}+(\frac{\left|\sigma_{s}\right|}{SFLS}{)}^{2}\geq 1$$

where $\sigma_{n}$ and $\sigma_{s}$ are the normal and shear stresses, respectively, on the contact surface, and NFLS and SFLS are the respective normal and shear failure strengths of the contact surface. The separation distance between contact points is the primary criterion for determining failure. As the separation distance gradually increases, the contact force between layers decreases proportionally. When the separation distance reaches the preset critical value CCRIT, it can be determined that the contact points have completely separated, resulting in delamination failure, where

(13) $$CCRIT=\frac{2E_{tie}}{S}$$

(14) $$S=\sqrt{\max(\sigma_{n},0{)}^{2}+{\left|\sigma_{s}\right|}^{2}}$$

where $E_{tie}$ is the interlayer failure energy release rate. Setting $E_{tie}=G_{\mathrm{II}c}$, $\sigma_{n}=0$, and $\sigma_{s}=SFLS$, CCRIT can be expressed as

(15) $$CCRIT=\frac{2G_{IIc}}{SFLS}$$

where $G_{\mathrm{II}c}$ is the energy release rate for Mode II. The interlayer interface stress is calculated as

(16) $$\tau^{0}=\sqrt{\frac{9\pi E_{S}G_{C}}{32N_{e}^{0}l_{e}}}$$

where *E_S_* is the transverse shear modulus, *G_C_* is the fracture energy release rate, $N_{e}^{0}$ is the number of elements in the cohesive zone, and $l_{e}$ is the element size. The parameters required for delamination failure simulation are shown in Table 5.

## 4. Results and Discussion

### 4.1. Result Validation

**Figure 5:**
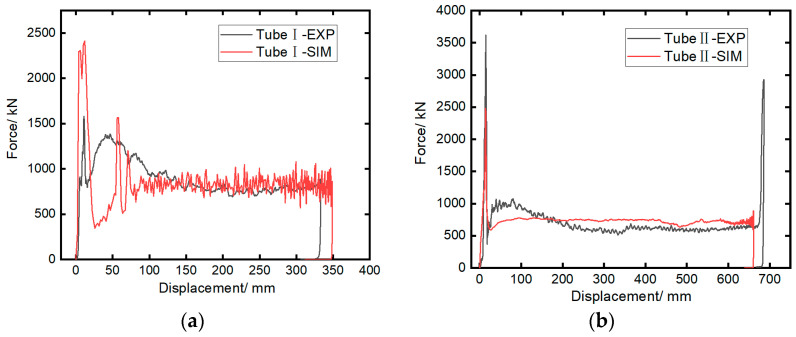
Axial crushing force–displacement curves from experiment and simulation: (**a**) Tube I; (**b**) Tube II.

**Figure 6:**
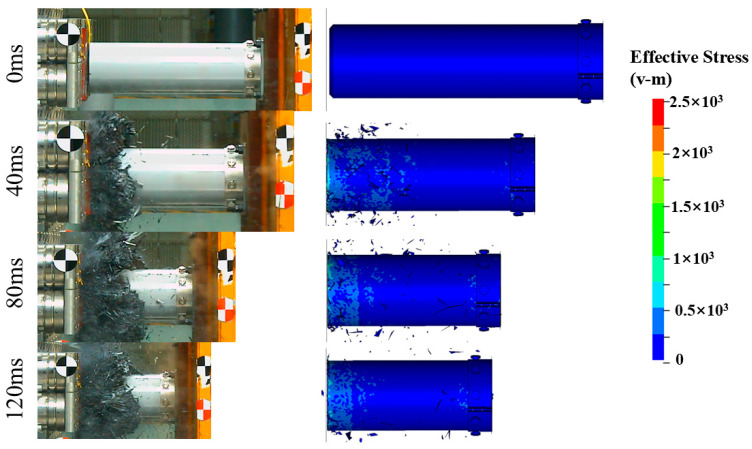
Crushing process of Tube I from experiment and simulation.

**Figure 7:**
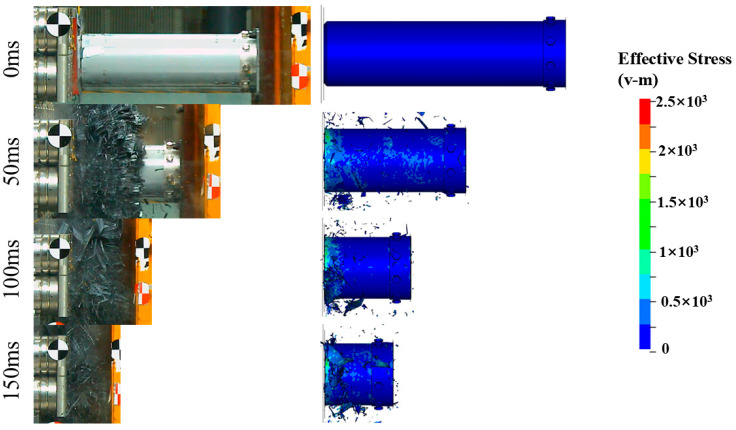
Crushing process of Tube II from experiment and simulation.

Figure 5 shows the impact force–displacement curves obtained from the experiments and simulations. It can be observed that the experimental results align well with the simulations. The impact response behaviors of Tubes I and II are similar [45,46]: after the specimen contacts the rigid wall, the impact force rises rapidly. The initial peak force of Tube II is 3617.8 kN, which is 128.8% higher than that of Tube I, due to the impact between the trigger device at the end of Tube II and the rigid wall. The impact force then quickly decreases to the plateau level and fluctuates slightly until the test ends. Compared to the folding deformation modes of similar structures made of aluminum alloy and stainless steel [47], the load–displacement curve of the CFRP structure in this study shows little fluctuation during the stable bearing stage, indicating a more stable failure process. This is verified by Figure 6 and Figure 7, where both composite tubes exhibit a progressive end failure mode, characterized by the stable deformation and failure of the tube wall material under the action of the rigid wall or trigger device, producing a large number of fragments.

Table 6 compares the crashworthiness indicators obtained from the experiments and simulations. The maximum error in the MCF and SEA between the experimental and simulation results is 4.8%, demonstrating the accuracy of the established model. In similar studies on CFRP structure crashworthiness, the SEA of CFRP structures with chamfered triggers typically ranges from 35 to 100 kJ/kg [48]. In comparison, the SEA of Tube I in this study is 38.69 kJ/kg, indicating considerable potential for improvement, but this value is still much higher than those of structures made from metals such as aluminum alloy (13.23 kJ/kg) and steel (10.01 kJ/kg) [47].

Compared to Tube I, the addition of a trigger device (Tube II) results in respective 21.2% and 21.9% reductions in the MCF and SEA. This phenomenon is consistent with previous findings [12], where the researchers compared the effects of three triggering methods—45° chamfered trigger, outward folding, and outward folding with cavities—on the impact response behavior of the structure. The reason for this is that the trigger device changes the failure mode of the structure, thereby affecting the energy absorption mechanism and crashworthiness. The specific reasons are discussed in Section 4.2.

### 4.2. Energy Absorption Mechanism

**Figure 8:**
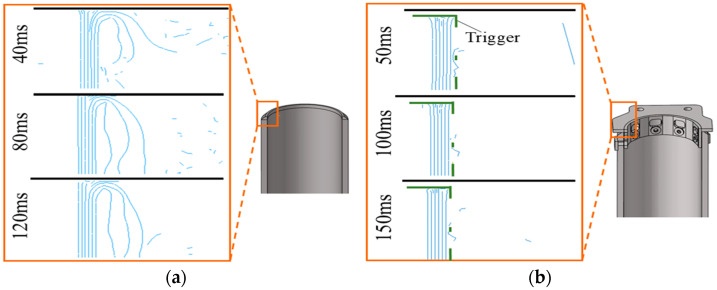
Cross-sectional view of tube wall during crushing: (**a**) Tube I; (**b**) Tube II.

Figure 8 shows cross-sectional views of the deformation of Tubes I and II. As observed in Figure 8a, the chamfer-triggered Tube I exhibits a “splaying” deformation mode, which is close to the failure mode of similar small-sized CFRP structures [21]. Initial cracks form at the impact end of the tube wall, leading to delamination failure at half the thickness of the tube wall material. During the loading process, the cracks propagate steadily along the impact direction, causing the tube wall material to bend inward and outward. Subsequently, friction occurs between the bent tube wall material and the rigid wall, leading to complex transverse shear failures inside the tube wall material, such as fiber breakage, matrix cracking, and delamination, eventually producing a large number of fiber fragments.

Compared to Tube I, the presence of the trigger device in Tube II restricts the initiation and propagation of inter-ply cracks in the tube wall, resulting in the consistent concentration of deformation and failure on the outside of the tube, ultimately leading to a deformation mode that differs entirely from that of Tube I. In this deformation mode, the energy absorption of the structure still occurs primarily through the crushing of the tube wall material and friction with the trigger device. However, insufficient damage to the tube wall material leads to a decrease in the structure’s load-bearing capacity, and a similar phenomenon has been observed previously [12].

**Figure 9:**
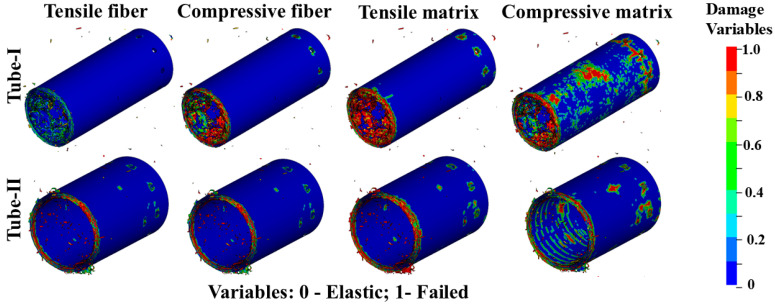
Damage distribution of composite material tubes.

Figure 9 shows the damage distribution of the tube wall materials of Tubes I and II at the same impact moment, including the fiber and the matrix in tension/compression. These variables can be output through the history variables in LS-PrePost, where a value of 0 indicates the material is in the elastic deformation stage, and a value of 1 indicates the element has failed. It can be seen that the tube wall material exhibits a coupling of multiple failure modes during impact, with the damage concentrated at the impact end of the tube wall. During the impact process, the failure of the matrix in tension/compression and fiber in compression occurs around the bolt holes.

## 5. Parameter Analysis

We investigate the effects of simulation parameters on the impact response of CFRP tubes using the Tube I model. The parameters include the failure parameters (DFAILT/DFAILC), the friction coefficient, and the interfacial strength (NFLS/SFLS). The calculation cases are shown in Table 7. In the naming of each case, F represents the friction coefficient, T is the maximum fiber failure strain, and S is the inter-ply interfacial strength. For example, “F1-T005-S1” indicates a static/dynamic friction coefficient (F) of 0.2/0.15, a maximum fiber failure strain (T) of 0.05/−0.05, and an inter-ply normal/tangential failure strength (S) of 50/75 MPa.

### 5.1. Failure Parameters

The calculation cases are set as F1-T02-S1, F1-T005-S1, and F1-T002-S1 to investigate the effects of fiber failure parameters on the impact response results. The maximum tensile/compressive failure strains (DFAILT/DFAILC) of the fibers are set in the three cases to 0.2/−0.2, 0.05/−0.05, and 0.02/−0.02, respectively; the static/dynamic friction coefficient is set to 0.2/0.15; and the inter-ply normal/tangential failure strength is set to 50/75 MPa.

**Figure 10:**
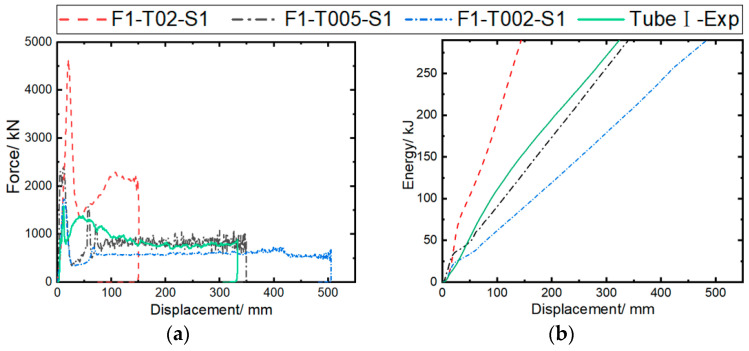
Comparison of experimental and simulated crushing responses of Tube I under different fiber failure parameters: (**a**) impact force–displacement curves; (**b**) energy absorption–displacement curves.

Figure 10 compares simulation and experimental results under different fiber failure parameters. It can be observed that the predicted load–displacement curves show consistent trends across all cases, indicating that the structure can stably bear the load. As the failure parameter increases from 0.02 (F1-T002-S1) to 0.2 (F1-T02-S1), the initial peak load and bearing capacity of the curve are significantly improved, resulting in the shortest crushing length of 150.3 mm with F1-T02-S1, and the longest crushing length of 505.6 mm with F1-T002-S1, under the same impact energy.

**Figure 11:**
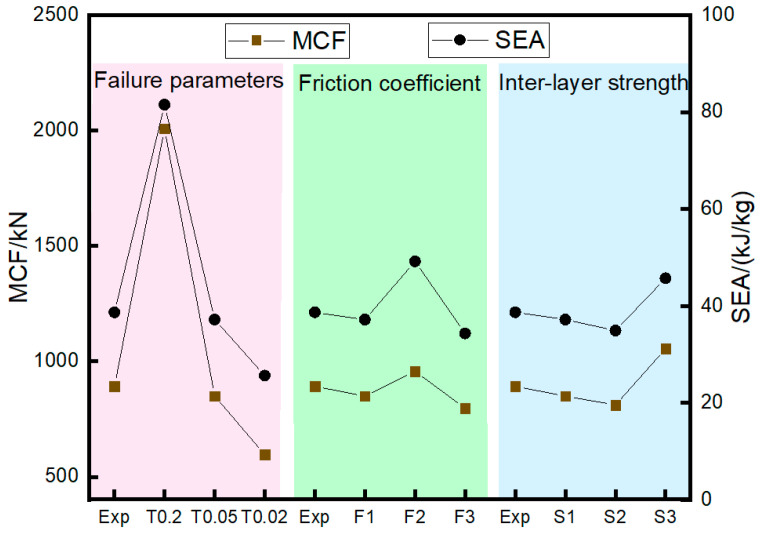
Effects of failure parameters (DFAILT/DFAILC), friction coefficient, and interfacial strength on MCF and SEA.

Figure 11 compares the predicted and experimental MCF and SEA. It can be observed that the MCF and SEA predicted by a maximum failure strain of 0.02 (F1-T002-S1) are 596.45 kN and 25.65 kJ/kg, respectively. As the maximum failure strain of the fiber increases from 0.02 to 0.2, MCF and SEA increase by 236.7% and 217.7%, respectively. Among them, the F1-T005-S1 case is the closest to the experimental results, with respective MCF and SEA errors of 4.8% and 3.9%.

**Figure 12:**
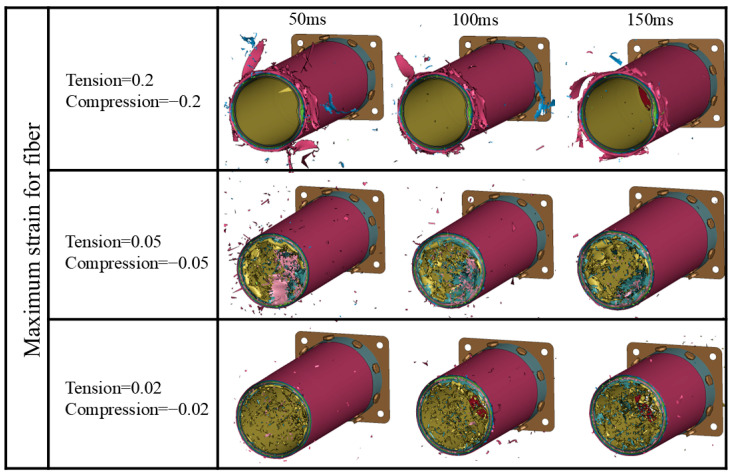
Crushing process of Tube I predicted using different failure parameters.

Figure 12 shows the axial crushing process of composite tubes at 50, 100, and 150 ms for different cases. It is observed that material deformation and failure are consistently concentrated at the tube end, consistent with the experimental process recorded by the camera in Figure 6. In the F1-T02-S1 case, the large fragments of the bent tube wall material easily cause the unstable failure of the structure during impact, as verified by the load–displacement curve in Figure 10a. As the maximum failure strain is reduced to 0.02, the fragment size of the tube wall material decreases, indicating a more stable failure process. However, due to premature element failure, the load-bearing capacity of the structure decreases. A comprehensive comparison shows that the deformation mode predicted in the F1-T005-S1 case is closer to the experimental results.

### 5.2. Friction Coefficient

**Figure 13:**
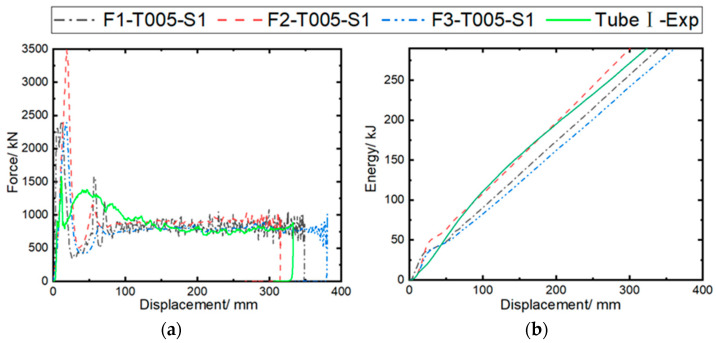
Comparison of experimental and simulated crushing responses of Tube I under different friction coefficients: (**a**) impact force–displacement curves; (**b**) energy absorption–displacement curves.

The calculation cases are set as F1-T005-S1, F2-T005-S1, and F3-T005-S1, with static/dynamic friction coefficients of 0.2/0.15, 0.4/0.3, and 0.1/0.08, respectively, to investigate the effect of the contact friction coefficient on the impact response results. The inter-ply normal/tangential failure strengths are set at 50/75 MPa, and the maximum tensile/compressive failure strain of the fiber with the highest prediction accuracy (0.05/−0.05) is used in the model. Figure 13 compares the predicted results of the impact force/energy–displacement curves under different friction coefficients. It can be observed that all predicted curves exhibit a trend similar to that of the experimental curve. As the dynamic/static friction coefficient increases from 0.1/0.08 to 0.4/0.3, the initial peak load of the curve increases significantly, while the load-bearing capacity is little affected.

Based on the analysis of Figure 11, the MCF and SEA predicted by the model (F3-T005-S1) with a friction coefficient of 0.1/0.08 are 797.18 kN and 34.33 kJ/kg, respectively. As the dynamic/static friction coefficient increases from 0.1/0.08 to 0.4/0.3, the MCF and SEA increase by 20.0% and 43.3%, respectively. This is mainly due to the enhancement of friction between the rigid wall and the bent plies, as well as the internal friction within the plies. The MCF and SEA predicted by the F2-T005-S1 model show the smallest errors (4.8% and 3.9%, respectively) compared to the experimental results.

**Figure 14:**
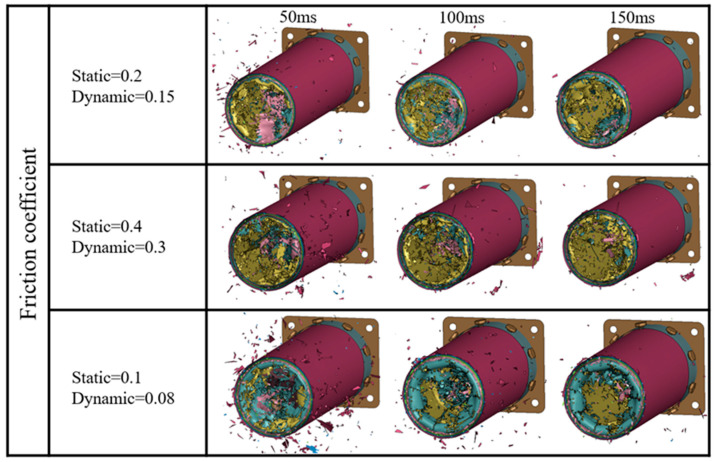
Simulated crushing process of Tube I under different friction coefficients.

Figure 14 illustrates the predicted axial crushing process of the composite tube at 50, 100, and 150 ms under different friction coefficients. It can be observed that the deformation modes predicted in all three cases exhibit progressive failure at the tube end. In the F2-T005-S1 case, the fiber bundles in the tube wall more easily break after experiencing large deformation. As the friction coefficient is reduced to 0.1/0.08, the size of the fiber bundles undergoing bending deformation increases. A comprehensive comparison indicates that the deformation mode predicted in the F1-T005-S1 case is closer to the experimental results.

### 5.3. Interfacial Strength

**Figure 15:**
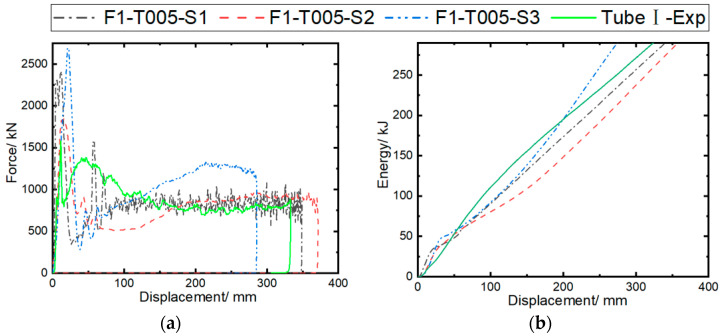
Comparison of experimental and simulated crushing responses of Tube I under different inter-ply failure stresses: (**a**) crushing force–displacement curves; (**b**) energy absorption–displacement curves.

The calculation cases are set as F1-T005-S1, F1-T005-S2, and F1-T005-S3 to investigate the effect of inter-ply interfacial strength on impact response results. NFLS and SFLS are set to 50/75, 25/45, and 21.3/30.4 MPa. The simulation parameters validated earlier are used in the model, with the maximum tensile/compressive failure strain of 0.05/−0.05 for the fibers, and a static/dynamic friction coefficient of 0.2/0.15. Figure 15 compares the predicted force/energy–displacement curves under different inter-ply interfacial strengths. The F1-T005-S1 case shows good consistency with the experimental curves, while the crushing forces of the F1-T005-S2 and F1-T005-S3 cases show significant fluctuations as the inter-ply property weakens. Figure 11 shows the errors between the simulated crashworthiness indicators and the experimental data. It can be observed that the MCF and SEA, as predicted in the F1-T005-S1 case, are 849.49 kN and 37.17 kJ/kg, respectively. As the normal/tangential interfacial strength is reduced from 50/75 MPa to 21.3/30.4 MPa, the MCF and SEA become 1054.7 kN and 45.69 kJ/kg, respectively. The F1-T005-S1 case results in the smallest errors in the MCF (4.8%) and SEA (3.9%) compared to the experimental results.

**Figure 16:**
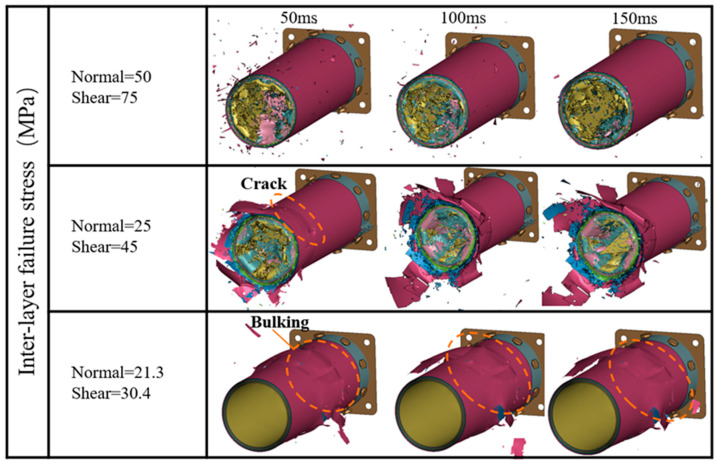
Simulated crushing process of Tube I under different inter-ply failure stresses.

Figure 16 shows the axial crushing process of the composite tube at 50 ms, 100 ms, and 150 ms under different interfacial strength coefficients. It can be observed that the F1-T05-S1 case exhibits progressive failure at the tube end. However, as the interfacial strength weakens, the tube in the F1-T05-S2 case develops circumferential cracks in the tube wall at 50 ms, and the curl size of the plies at the incident end of the tube wall is larger than in the F1-T05-S1 case. The tube in the F1-T05-S3 case, which has the weakest interfacial property, mainly shows buckling deformation, and no damage occurs at the tube end, indicating that interfacial strength has a significant effect on the failure mode.

## 6. Conclusions

The structural characteristics of the energy-absorbing components of high-speed trains were analyzed. CFRP circular tube structures with a thickness of 18 mm were prepared using the fiber winding process. Using sled impact tests and numerical simulations, the impact response and crashworthiness of CFRP tubes with chamfered and embedded triggers were investigated. A sensitivity analysis of the modeling parameters was conducted using the validated finite element model. Our main conclusions are as follows:

1. The energy-absorbing structures exhibit stable deformation and failure modes under the cases of both a chamfered trigger (Tube I) and embedded trigger (Tube II). The MCF and SEA of Tube I are 891.89 kN and 38.69 kJ/kg, respectively, which, in comparison, are reduced in Tube II by 21.2% and 21.9%;

2. The established finite element model can effectively predict the impact response behavior of CFRP circular tubes, and the errors between the predicted MCF and SEA indicators and the experimental results are less than 5%;

3. Parametric studies showed that the predicted MCF and SEA were sensitive to the failure parameters (DFAILT/DFAILC), the friction coefficient, and the interfacial strength (NFLS/SFLS). At the same time, the crushing failure modes of the composite tubes were stable regardless of the variation in these parameters.

In summary, the proposed CFRP tubes displayed excellent MCF and SEA, indicating the feasibility of lightweight energy-absorbing components. Adjusting the triggering mechanism acts as an effective approach to improving the load-carrying capability. The Structural parameter optimization for the triggering devices will be performed in our future work.

## Figures and Tables

**Table 1:** Ply and structural parameters of composite tubes.

Code	*D*/mm	*L*/mm	*T*/mm	Layer		Mass/kg
				Thickness/mm	Layers	
Tube I	220	914	18	0.2	90	21.26
Tube II	220	914	18	0.2	90	21.27

**Table 2:** Mechanical parameters of CFRP material.

Parameters	Name	Value
Mass density	ρ/(g/cm^3^)	1.60
Young’s modulus, longitudinal direction Young’s modulus, longitudinal direction	$E_{a}$/GPa $E_{b}$/GPa	153
		13.73
Longitudinal tensile strength Transverse tensile strength	$X_{t}$/MPa $Y_{t}$/MPa	2537
		155.21
Longitudinal compressive strength Transverse compressive strength	$X_{c}$/MPa $Y_{c}$/MPa	1580
		34.7
Shear modulus	$G_{ab}$/GPa	3700
Shear strength	$S_{c}$/MPa	90

**Table 3:** Failure parameters of CFRP material model.

Parameters	Name	Value
Maximum strain for matrix straining	DFAILM	0.8
Maximum tensorial shear strain	DFAILS	0.074
Shear stress parameter	ALPH	0.1
Softening for fiber tensile strength	FBRT	0.1
Reduction factor for compressive fiber strength	YCFAC	5.165
Maximum strain for fiber tension	DFAILT	0.02
Maximum strain for fiber compression	DFAILC	−0.02
Effective failure strain	EFS	0.6

**Table 4:** Parameters of Q355 material.

Parameters	Name	Value
Mass density	ρ/(g/cm^3^)	7.85
Young’s modulus	E/GPa	120
Poisson’s ratio	ν	0.3
Yield stress	SIGY/MPa	355
Tangent modulus	ETAN/MPa	2100

**Table 5:** Material parameters required for delamination failure simulation.

Parameters	Name	Value
Normal failure stress	NFLS/MPa	50
Shear failure stress	SFLS/MPa	75
Static friction coefficient	FS	0.2
Dynamic friction coefficient	FD	0.15
The critical distance at which the PARAM interface completely fails	CCRIT/mm	0.15675

**Table 6:** Comparison of experimental and simulated crashworthiness indicators.

Sample	MCF (kN)		Error	EA (kJ)		Error	SEA (kJ/kg)		Error
	EXP	SIM		EXP	SIM		EXP	SIM	
Tube I	891.89	849.49	4.8%	297.00	301.76	1.6%	38.69	37.17	3.9%
Tube II	703.02	730.62	3.9%	482.31	486.65	0.9%	30.20	31.40	3.4%

**Table 7:** Experimental and simulation results of Tube I with different simulation parameters.

Code	Static/Dynamic Friction Coefficient	DFAILT/DFAILC	NFLS/SFLS (MPa)	MCF		SEA	
				SIM (kN)	Error	SIM (kJ/kg)	Error
F1-T02-S1	0.2/0.15	0.2/−0.2	50/75	2008.25	125.2%	81.50	100.6%
F1-T005-S1	0.2/0.15	0.05/−0.05	50/75	849.49	4.8%	37.17	3.9%
F1-T002-S1	0.2/0.15	0.02/−0.02	50/75	596.45	33.1%	25.65	33.7%
F2-T005-S1	0.4/0.3	0.05/−0.05	50/75	956.83	7.3%	49.19	27.1%
F3-T005-S1	0.1/0.08	0.05/−0.05	50/75	797.18	10.6%	34.33	11.3%
F1-T005-S2	0.2/0.15	0.05/−0.05	25/45	811.33	9.0%	34.92	9.7%
F1-T005-S3	0.2/0.15	0.05/−0.05	21.3/30.4	1054.70	18.3%	45.69	18.1%

## Data Availability

The original contributions presented in the study are included in the article, further inquiries can be directed to the corresponding author.
